# Characterization and phylogenetic analysis of the complete mitochondrial genome of pathogen *Trichosporon inkin* (Trichosporonales: Trichosporonaceae)

**DOI:** 10.1080/23802359.2021.1882912

**Published:** 2021-03-11

**Authors:** Qiaofeng Liu, Xin Wang

**Affiliations:** a Department of Pathology and Pathophysiology, Chengdu Medical College, Chengdu, China; b Department of Pathogenic Biology, Chengdu Medical College, Chengdu, China

**Keywords:** Pathogen, mitochondrial genome, phylogenetic analysis, evolution

## Abstract

In the present study, the complete mitochondrial genome of *Trichosporon inkin* was sequenced and assembled. The complete mitochondrial genome of *T. inkin* contained 22 protein-coding genes (PCG), 2 ribosomal RNA (rRNA) genes, and 24 transfer RNA (tRNA) genes. The total size of the *T. inkin* mitochondrial genome is 39,466 bp, with the GC content of 27.56%. Phylogenetic analysis based on combined mitochondrial gene dataset indicated that the *T. inkin* exhibited a close relationship with *Trichosporon asahii*.

The genus *Trichosporon* is a group of yeast-like fungi, living widespread in nature (Marine et al. 2015; Duarte-Oliveira et al. 2017). Over 50 species have been assigned into this genus (Colombo et al. 2011; Gouba et al. 2014). Most species from this genus are classical opportunistic pathogens, which reside harmlessly as commensals on the skin and the gastrointestinal tract of healthy individuals (Zhang et al. 2011; Guo et al. 2018). Species identification of *Trichosporon* can be used to trace the origin of nosocomial infection and the hospital or regional specificity of isolated strains (Sun et al. 2012; Guo et al. 2018). Mitochondrial genomes have been widely used in the phylogenetic analysis of basidiomycete species (Li et al. 2019b; Li, He, et al. 2020; Wang, Song, et al. 2020). However, up to now, only one complete mitochondrial genome from the genus *Trichosporon* has been reported (*Trichosporon asahii*). The mitochondrial genome of *Trichosporon inkin* will promote the understanding of the phylogeny, origin, and taxonomy of this important genus.

The specimen (*T. inkin*) was collected from a Hospital in Sichuan, China (104.05 E; 30.65 N) in 2017. The *T. inkin* was isolated from the cultures of diseased foot tissue and identified by deoxyribonucleic acid sequencing. We stored the specimen in Culture Collection Center of Chengdu Medical College (No. Trisp_x3). The complete mitochondrial genome of *T. inkin* was sequenced and *de novo* assembled according to previously described methods (Li, Liao, et al. 2018; Li, Xiang, et al. 2019; Wang, Song, et al. 2020). Briefly, we extracted the total genomic DNA of *T. inkin* for sequencing using a Fungal DNA Kit D3390-00 (Omega Bio-Tek, Norcross, GA, USA). We then purified the extracted genomic DNA using a Gel Extraction Kit (Omega Bio-Tek, Norcross, GA, USA). The purified DNA was stored in Chengdu Medical College (No. DNA_Trisp_x3). Sequencing libraries were constructed using a NEBNext® Ultra™ II DNA Library Prep Kit (NEB, Beijing, China). The Illumina HiSeq 2500 Platform (Illumina, San Diego, CA) was used to conduct whole genomic sequencing (WGS) of *T. inkin*. The mitochondrial genome of *T. inkin* was *de novo* assembled using SPAdes 3.9.0 (Bankevich et al. 2012; Li, Ren, et al. 2020) with a kmer size of 17. Since organelle sequences usually have more copies than nuclear gene sequences, the mitochondrial genome we finally obtained showed high coverage. In addition, MITObim (Hahn et al. 2013), and NOVOPlasty (Dierckxsens et al. 2017) were also used to test the assembly of this study. All the software obtained complete mitogenomes identical to this study, which proves that the mitochondrial genome obtained in the present study is reliable.

We annotated the complete mitochondrial genome of *T. inkin* according to previously described methods (Li, Chen, et al. 2018; Li, Wang, et al. 2018). Briefly, the protein-coding genes, rRNA genes, tRNA genes, and introns of the *T. inkin* mitogenome were initially annotated using MITOS (Bernt et al. 2013) and MFannot (Valach et al. 2014), both based on the genetic code 4. The PCGs and rRNA genes were further annotated according to methods described previously (Wang, Jia, et al. 2020; Wang, Wang, et al. 2020; Wu et al. 2021). The tRNA genes in the *T. inkin* mitogenome were also predicted with tRNAscan-SE v1.3.1 (Lowe and Chan 2016). Introns in the *T. inkin* mitogenome were named according to previous studies (Zhang and Zhang 2019).

The complete mitochondrial genome of *T. inkin* is 39,466 bp in length. The base composition of the *T. inkin* mitogenome is as follows: A (36.17%), T (36.27%), G (13.99%), and C (13.57%). The complete mitochondrial genome of *T. inkin* contains 22 protein-coding genes, 2 ribosomal RNA genes (*rns* and *rnl*), and 24 transfer RNA genes. Nine introns were detected in the *T. inkin* mitogenome, including 4 in the *cox1*, 2 in *cox2*, 1 in *cob*, *nad5*, and *rnl* genes, respectively. These introns all belonged to the Group I. Introns in PCGs of the *T. inkin* mitogenome were named according to previous studies (Zhang and Zhang 2019), including Tin.cox1P386, Tin.cox1P709, Tin.cox1P807, Tin.cox1P867, Tin.cox2P318, Tin.cox2P685, Tin.nad5P717, and Tin.cobP506. Six intronic ORFs were detected in the *T. inkin* mitogenome, which encoded LAGLIDADG endonucleases. We constructed a phylogenetic tree for 18 basidiomycete species to investigate the phylogenetic status of *T. inkin*. *Rhizopogon salebrosus* from the order Boletales was set as an outgroup (Li, Ren, et al. 2019). The Bayesian analysis (BI) method was used to construct phylogenetic tree based on the combined 14 core protein-coding genes according to previously described methods (Li, Wang, et al. 2019; Li et al. 2019a; Li, Yang, et al. 2020). As shown in the phylogenetic tree (Figure 1), the mitochondrial genome of *T. inkin* exhibited a close relationship with that of *T. asahii* (JH925097).

**Figure 1.**
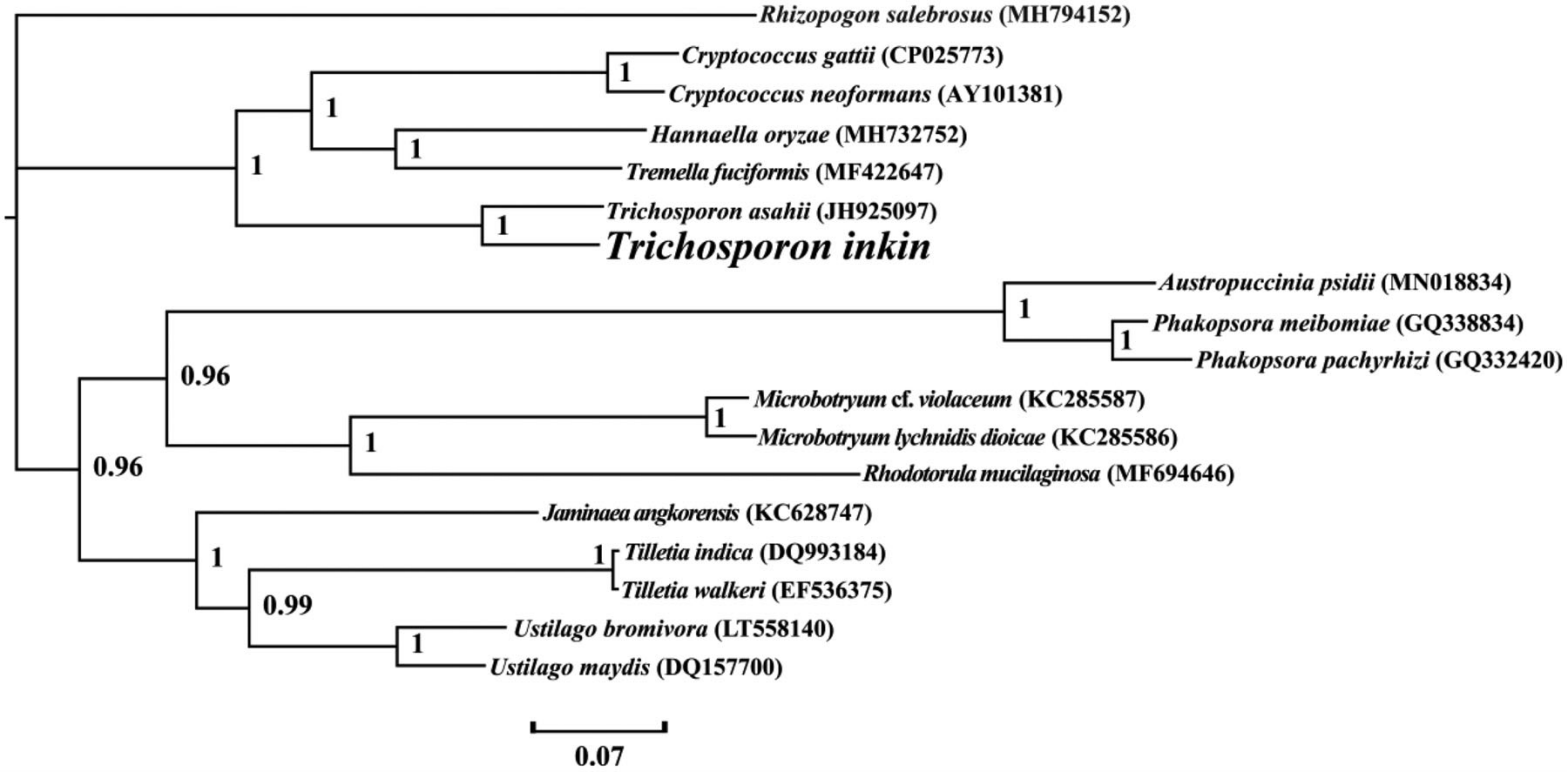
Bayesian phylogenetic analysis of 18 species based on the combined 14 core protein-coding genes. Support values are Bayesian posterior probabilities (BPP). Accession numbers of mitochondrial sequences used in the phylogenetic analysis are listed in brackets after species.

## Data Availability

This mitogenome of *Trichosporon inkin* was submitted to GenBank under the accession number of MT801082. (https://www.ncbi.nlm.nih.gov/nuccore/ MT801082).
